# Ensemble inference by integrative cancer networks

**DOI:** 10.3389/fgene.2014.00059

**Published:** 2014-03-31

**Authors:** Antonio Mora, Monia Taranta, Nazar Zaki, Elarbi Badidi, Caterina Cinti, Enrico Capobianco

**Affiliations:** ^1^Laboratory of Integrative Systems Medicine, Institute of Clinical Physiology, CNRPisa, Italy; ^2^Bioinformatics Lab, College of Information Technology, United Arab Emirates UniversityAl Ain, UAE; ^3^Laboratory of Experimental Oncology, Institute of Clinical Physiology, CNRSiena, Italy; ^4^Center for Computational Science, University of MiamiMiami, FL, USA

**Keywords:** networks, modularity, predictive inference, cancer markers, epigenetic therapy

## Background

Cancer is a multifactorial disease with a striking heterogeneity due to genetic, epigenetic and transcriptional changes involving a myriad of genes and proteins. While these factors are relevant to clinical prognosis and medical treatment, a system's approach is needed to unravel the complexities underlying intertwining carcinogenesis mechanisms. In particular, networks allow for straightforward integration of molecular, genetic, clinical, and topological features embedded in measurable cancer data. Modeling such data leads to an assessment of significant changes in conditions which affect the cellular mechanisms, in particular dysregulating them. Ultimately, treatment of cancer as a systems disease indicates a challenging translation from systems biology to systems medicine. Markers are key players in cancer, characterized by the reference entity (gene, protein, etc.,) and by their individual or composite nature. We aim to show that the association of markers with detected network modules presents advantages compared to the consideration of individual markers.

Network complexity can be characterized in many possible ways, and both the specific data and the network structure represent factors conditioning any possible inference approach. The structural organization of networks is measurable at both local and global network scale. Consider for instance node degree and link density as a starting point, then move to the analysis of degree-degree correlation, and finally to the exploration of modularity (core/community structure). While such translation allows for validating the presence of non-random network dynamics, the role of stochasticity suggests that a network can be conceived as an example of an ensemble of networks with certain structural properties, i.e. a sort of example sampled from a network space. Notably, by focusing on the structure of networks, and not on the dynamics defined on them, the concept of stationarity is simplified by considering the fact that despite natural networks arise often from non-equilibrium processes, the notion of equilibrium investigated through the previously described translation (roughly speaking, single nodes—correlated nodes—modules and cross-linked modules) can be considered an abstraction within a frame in which network ensembles are stationary entities and each example or component network can be seen as a state of the system.

Markers involve several complex phases, such as: discovery, identification, and validation. Networks offer an interesting opportunity with regard to the study of markers: they allow to establish their relevance as individual entities and also as components of a cluster or module. Supported by recent literature (Dao et al., 2011; Peer and Hacohen, 2011; Bebek et al., 2012; Wu et al., 2012; Ben-Hamo and Efroni, 2013), we hypothesize that by switching their role, from individual to team players, markers may provide novel information on cancer, especially when studied in a pathway context. In particular, markers examined at a network scale may reveal their systems relationships, generating synergistically active candidates. This fact is important as it bypasses limitations due to low reproducibility between differential expression (DE) studies, because of the cellular heterogeneity within a tissue, genetic heterogeneity among patients, and other reasons (Ein-Dor et al., 2005, 2006). Chuang et al. (Chuang et al., 2007) highlight that sub-networks, i.e., connected components in a protein interaction network, which are induced by markers, show superior reproducibility compared to isolated gene markers. Also, genes with known breast cancer mutations may be not detected by DE studies, but still play an important role in interconnecting DE genes. Sub-networks were detected based on the maximization of the mutual information computed between the activity scores (averaged normalized gene expressions) and the disease status (metastasis/non-metastasis). Similarly, Lee et al. (2008) computed the activity of pathways through a related score corresponding to the activity of the subset of genes in each pathway (called CORGs) found to better discriminate the disease status. Notably, we looked carefully at the new generation of pathway enrichment tools in the bioinformatics literature, and selected for our analyses *GeneMANIA* (Mostafavi et al., 2008; Warde-Farley et al., 2010). This tool integrates known co-expression, co-localization, pathway, protein interaction and genetic interaction relationships to the DE gene list, and predicts from the latter additional genes, with the result of strengthening the functional enrichment analysis. This integrative omics approach becomes a binder of a wide range of biological information layers in the same meta-network, thus including many possible generators of marker modules. Inclusion of information from non-DE genes is also possible if it is important in terms of connectivity, and considered into the analysis through over-representation or scoring techniques.

The question addressed in this Opinion therefore is: how effective is an integrative approach and the additional inference power made available for our understanding of the role of cancer markers? In parallel studies that we are conducting, and whose results are centered on treatment-specific profiling and pathway annotation, we have performed functional enrichment analysis of multi-drug resistant osteosarcoma (MDR-OS) cells from the HosDXR150 cell line after three epigenetic treatments working against drug resistance (Esteller, 2007; Bock, 2012). The same data source is used here to perform a second-generation analysis, following the meta-network approach. The mechanism we want to study is in the realm of epigenetic therapy, and consists of a de-methylating agent (5-Aza-dC, DAC), a de-acetylating agent (TSA), and a treatment combining both. We hypothesize that our inference approach can shed light over the impact on cells of single versus combined epigenetic treatments, by identifying module-specific and module-shared markers at systems scale.

## Methods

### Network and module marker generation

cDNA microarray analysis was performed to provide expression measurements of 1920 genes of MDR-OS cells after the three treatments (details in Supplementary File Experiment.doc). The gene IDs of the DE genes after each treatment were fed to the *GeneMANIA* web tool (http://www.genemania.org), and we explored co-expression, genetic interaction, co-localization, pathway and physical interaction network data. Edge weighting was based on GO–Biological Processes. The integrated networks (IN) were generated from only the DE genes and by adding the 20 most closely related genes, according to the algorithm. The procedure was repeated for all cell treatments, forming 6 networks; a common hypergeometric over-representation test (*p*-value = 0.05) determined enriched pathways and functional modules, and those sharing the same group of genes were merged into a single group, then used as module markers.

## Results and discussion

### Gene sets and networks

The treatment with the de-methylating agent DAC produced 57 genes significantly up-regulated, and 69 down-regulated. The treatment with the de-acetylating agent TSA produced 40 genes significantly up-regulated, and 68 down-regulated. The combined treatment with DAC + TSA produced 16 genes significantly up-regulated, and 46 down-regulated. Those gene lists were fed to *GeneMANIA*. Three of the six produced networks were generated using the original DE genes (one for the DAC treatment, one for TSA, and one for the combined DAC + TSA), while the other three networks were generated by extending to additional predicted 20 nodes in each network, i.e., highly connected genes in the IN (one for each treatment type). For the DAC treatment, 60% of the genes *de novo* connected within the IN were present in the microarray, but were not DE genes, while the remaining 40% corresponded to genes not present in the microarray (PDGFRB, PIK3R1, MAPK3, EGF, CASP9, GSK3B, STAT5A, and MAP2K2). Exactly the same situation was observed for the TSA treatment, where 40% of the added genes were not in the microarray (PIK3R1, MAPK3, MAP2K2, CHUK, PIK3R2, IKBKB, PIK3CB, and TRAF2). For the combined treatment, only 5 new genes (25%) were added (IL12B, IL10, CHUK, MAPK9, and IKBKB). Intuitively, the extension of the network to highly connected genes in an abstract space of data multitude suggests a possible recovery of potentially important genes excluded from the experiment. In turn, the predictive inference approach can generate more testable hypotheses centered on the possible role of markers.

### Module markers

A module marker is a group of genes with some detected properties, beyond their simple collection. A module is considered “active” if DE genes are included, while a more specific characterization involves gene connectivity, considering cases of network of integrated interaction, pathway, co-expression, co-localization, and genetic interaction data. Such group of genes may form a sub-network involving one or multiple pathways, or functional modules, over-represented in one or more of those. Following this idea, the 126 DE genes after DAC treatment yielded 240 module markers, the 108 DE genes after TSA treatment yielded 161 module markers, and the 62 DE genes after DAC + TSA treatment yielded 207 module markers. Generating module markers from an IN may offer advantages over the use of only interactions or pathways; an advantage refers to selecting specific or multiple data types. A data multitude is represented in the Supplementary File *Figure.doc* (panel A), with three out of six module markers appearing after DAC treatment, and other three after TSA treatment. A combination of co-expression (emphasized in purple), pathway (blue), interaction (red), co-localization (gray), predicted interaction (yellow), or genetic interaction (green) characterizes the modules, while the removal of any specific data type would affect the IN's integrity. A limited data type variety is behind the Supplementary File *Figure.doc* (panel B): two modules (b and f) come from co-expressions, one (a) comes mainly from pathways, and the rest from both. The other edges (interactions, predicted interactions and co-localization) only appear in a few cases, implying that their removal would not affect the connectivity of the PIN.

### Inter-treatment comparisons

**Figure 1:**
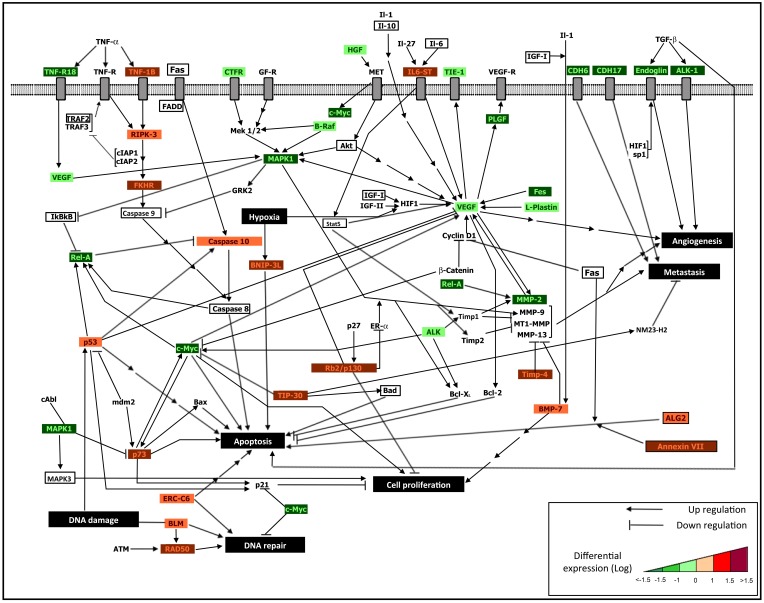
**An integrative regulatory map**. Integrated interactomic relationships are presented, starting from the DE genes derived from the cDNA microarray analysis, and considering connectivity with non-DE genes (marked in bold font) obtained from the reported network extension and functional enrichment tools. The non-DE genes excluded from the experiments (labeled without colors) allow to establish systems relationships with DE genes (down-regulated after treatment, which appear in green frame, and up-regulated after treatment, which appear in red frame), and to highlight the biological influence of epigenetic treatments. We identified major influences in terms of: activation of osteoblast differentiation and apoptotic signaling, and inhibition of cell proliferation, metastasis and angiogenesis. In particular, following the described treatments, the re-expression of epigenetically silenced key genes re-establishes cellular homeostasis throughout mechanisms such as: 1. Osteoblast differentiation (i.e., IL-6, IL6ST, IGF1, TIMP4, TIMP1, BMP-7, all emerging from combined treatment); 2. Drug sensitivity of MDR-OS cells through the re-activation of both extrinsic apoptotic signaling (i.e., TNF-1B, RIPK-3, FAS, FADD, genes indicated with red frame and emerging from combined treatment) and intrinsic apoptotic signaling (i.e., ALG2, P53, P73, CASP10, ERCC6, BAX, BAD, BNIP-3L, genes indicated with red frame and emerging from DAC and TSA single treatments, as illustrated in Table 1); 3. Inhibition of cell proliferation, angiogenesis and metastasis by down-regulation of some genes (i.e., VEGF, MAPK1, C-MYC, REL-A, MMP-2, genes indicated with green frame), as a consequence of re-expression of epigenetically modified genes (indirect treatment's influence). The integrated approach allows to better decipher the complex cellular mechanisms which led the tumor cells to acquire the multi-drug resistance phenotype and a pro-survival advantage, therefore identifying tumor-specific markers useful to future targeted therapy.

Table 1 (top) shows 9 out of 126 genes expressed after DAC treatment which are also expressed after co-treatment, and 20 out of 108 genes expressed after TSA which are expressed after the co-treatment. When comparing module markers generated from the DE genes, no pathway is conserved between single and combined treatments. However, things change with the extended gene set. The number of conserved genes between treatments slightly increases: 13 out of 126 DE genes appeared both in DAC and in co-treatment, while 25 out of 108 DE genes appeared both in TSA and in co-treatment. Three of the four additional genes in the first case (IL6, RELA, IFNB1) were genes expressed in DAC but not in DAC + TSA, while the fourth gene (AKT1) was not DE. Similarly, RELA was in TSA but not in DAC + TSA, while the other four genes (FADD, AKT1, CASP8, NFKB1) were not expressed in either TSA or co-treatment. An interesting result appears in column 4. Besides the modest increase in conserved genes, results show a substantial increment in the number of conserved module markers when using IN. In this case, 24 module markers are conserved in DAC and DAC + TSA, while 26 module markers are conserved between TSA and DAC + TSA (see Supplementary Table, part A and B, respectively). This evidence naturally depends on the types of treatments which are available, but opens for the possibility of assessing whether conserved module can be considered persistent between treatments of different nature. Overall, this approach suggests the opportunity of running a certain experimental design, and through module markers it emphasizes whether also indirect effects should be accounted through the embedded connectivity. Table 1 (middle) shows some interesting conserved module markers for both cases, while Table 1 (bottom) shows module markers specific to co-treatment. Notably, the DE genes after DAC and TSA present 13 gene markers in common, but 42 module markers in common under after the extension. As an example of integrative analysis, Figure 1 reports a map of gene and pathway regulation under the influence of the described treatments, single and combined (Table 1). The *Supplementary Table* file reports lists of gene and marker modules.

Table 1**Single gene marker versus module marker across epigenetic treatments**.

**Table:** 

**Treatment type**	**Source: DE genes**		**Source: extended gene list**	
	**Gene markers**	**Induced module marker**	**Gene markers**	**Induced module markers**
From DAC to DAC + TSA	9	0	13	24
From TSA to DAC + TSA	20	0	25	26

**Table:** 

**Module marker ID**	**# Pathways enriched in module**	**Best pathway**	**FDR –best pathway**	**# Genes in module marker**	**# Genes in pathway**	**Genes**
7	1	T cell activation	9.97e-08	15	217	AKT1, CD3G, CD47, CD8B, IFNB1, IGF1, IL6, IL6ST, INS, KIT, MYB, PIK3CA, **PIK3R1**, TNFSF18, WNT1
13	1	JAK-STAT cascade	1.04e-06	10	89	**EGF**, IGF1, IL6, IL6ST, KIT, MAPK1, **MAPK3**, SH2B2, **STAT5A**, STAT5B
52	1	Growth factor receptor binding	2.03e-04	7	70	**EGF**, IL6, IL6ST, PDGFB, PDGFRA, **PDGFRB**, TGFAlfa
74	2	Apoptotic signaling pathway	9.42e-04	9	173	AKT1, **CASP9**, CDKN1A, IGF1, PDCD6, SFN, TOPORS, TP53, TP73
83	4	Regulation of smooth muscle cell proliferation	1.15e-02	4	35	IGF1, IL6, PDGFB, **PDGFRB**
122	1	Cytokine receptor binding	7.88e-03	7	139	CXCL13, ENG, IFNB1, IL6, IL6ST, **PIK3R1**, TNFSF18
127	4	Regulation of cysteine-type endopeptidase activity involved in apoptotic process	8.46e-03	7	142	AKT1, **CASP9**, CD27, IFNB1, MYC, SFN, XDH
18	9	TRIF-dependent toll-like receptor signaling pathway	3.27e-07	9	67	**CHUK**, IKBKB, **MAP2K1**, **MAP2K2**, MAPK1, **MAPK3**, NFKB1, NFKBIA, RELA
21	2	Apoptotic signaling pathway	7.05e-07	12	173	AKT1, BAD, CASP8, CD38, CFLAR, FADD, HGF, HTT, PDCD6, RIPK3, TP53, **TRAF2**
22	1	T cell activation	7.98e-07	13	217	ADAM17, AKT1, LCK, LCP1, MALT1, NCKAP1L, PIK3CA, **PIK3CB**, **PIK3R1**, **PIK3R2**, RAG1, TRAF2, TCRB
29	1	Regulation of cytokine production	2.76e-06	14	296	ADAM17, **CHUK**, FADD, **IKBKB**, INHA, MALT1, NCKAP1L, NFKB1, NFKBIA, RELA, RIPK3, TAX1BP1, **TRAF2**, UBA7
32	2	regulation of type I interferon production	3.90e-06	8	67	**CHUK**, **IKBKB**, NFKB1, NFKBIA, RELA, RIPK3, TAX1BP1, UBA7
33	2	Regulation of cysteine-type endopeptidase activity involved in apoptotic process	8.80e-06	10	142	AKT1, BAD, CASP8, FADD, HGF, LCK, RAF1, TNFSF15, **TRAF2**, VEGFA
38	2	I-kappaB kinase/NF-kappaB cascade	8.92e-05	10	186	BMP7, CASP10, CASP8, CFLAR, **CHUK**, FADD, **IKBKB**, MALT1, NFKBIA, RELA
73	1	B cell activation	5.74e-03	6	95	ADAM17, CD38, GPR183, INHA, NCKAP1L, RAG1

**Table:** 

**Module marker ID**	**# Pathways enriched in module**	**FDR –best pathway**	**# Genes in module marker**	**# Genes in pathways**	**Genes**
85	Necrotic cell death	4.78e-05	4	14	FADD, FAS, FASLG, TNF
120	Regulation of osteoclast differentiation	5.72e-04	4	27	IFNG, **IL12B**, IL4, TNF
167	Leukocyte apoptotic process	2.05e-03	4	40	AKT1, AXL, **IL10**, IL2
237	Lymphocyte apoptotic process	8.98e-03	3	24	AKT1, **IL10**, IL2
306	B cell apoptotic process	4.14e-02	2	10	**IL10**, IL2

Top: Gene and Module Markers comparisons under various treatments.

Mid: Annotation of Conserved module markers between DAC and DAC − TSA treatments.

Annotation of Conserved module markers between TSA and DAC − TSA treatments.

Bottom: Examples of annotated module markers specific to co-treatment (DAC + TSA).

^*^Listed in bold font in the last column examples of extended genes, i.e., missing in the microarray but found connected in the network by the described method.

### Final remarks

Epigenetics implies heritable changes in gene expression without involvement of DNA sequence. Gene silencing is a complex biological process which involves methylation, and leads to disease development once dysregulated. The high frequency of epigenetic changes in cancer has motivated research into new therapeutic approaches aimed to reverse gene silencing. DNA methylation inhibitors, together with histone deacetylase inhibitors, are examples of valid drug targets conceived toward the re-activation of silenced genes. Future avenues include activation of single genes by exploiting single agents or also the combination of epigenetic drugs, thus emphasizing the synergistic activities between DNA methylation and HDAC inhibitors, and considering likely non-specificity in terms of gene re-activation. The identification of modules at the network scale leads to an integrative systems approach which goes beyond single marker analysis and exploits synergistic marker dynamics in support of combinatorial experiments. Our preliminary results show that the recovery of latent connectivity may re-position the markers depending on the module-integrated biodata multitude and on the nature of the edges linking the nodes.

## References

[B1] Bebek G. Koyutürk M. Price N. D. Chance M. R. (2012). Network biology methods integrating biological data for translational science. Brief. Bioinform. 13, 446–459 10.1093/bib/bbr075 22390873 PMC3404396

[B2] Ben-Hamo R. Efroni S. (2013). Networks as biomarkers. Syst. Biomed. 1, 34–41 10.4161/sysb.26474

[B3] Bock C. (2012). Analysing and interpreting DNA methylated data. Nat. Rev. Genet. 13, 705–719 10.1038/nrg3273 22986265

[B4] Chuang H.-Y. Lee E. Liu Y.-T. Lee D. Ideker T. (2007). Network-based classification of breast cancer metastasis. Mol. Syst. Biol. 3, 140 10.1038/msb4100180 17940530 PMC2063581

[B5] Dao P. Wang K. Collins C. Ester M. Lapuk A. Sahinal S. C. (2011). Optimally discriminative subnetwork markers predict response to chemotherapy. Bioinformatics 27, 1205–1213 10.1093/bioinformatics/btr245 21685072 PMC3117373

[B6] Ein-Dor L. Kela I. Getz G. Givol D. Domany E. (2005). Outcome signature genes in breast cancer: is there a unique set? Bioinformatics 21, 171–178 10.1093/bioinformatics/bth469 15308542

[B7] Ein-Dor L. Zuk O. Domany E. (2006). Thousands of samples are needed to generate a robust gene list for predicting outcome in cancer. Proc. Natl. Acad. Sci. U.S.A. 103, 5923–5928 10.1073/pnas.0601231103 16585533 PMC1458674

[B8] Esteller M. (2007). Cancer epigenomics: DNA methylomes and histone-modification maps. Nat. Rev. Genet. 8, 286–298 10.1038/nrg2005 17339880

[B9] Lee E. Chuang H.-Y. Kim J.-W. Ideker T. (2008). Inferring pathway activity toward precise disease classification. PLoS Comput. Biol. 4:e1000217 10.1371/journal.pcbi.1000217 18989396 PMC2563693

[B10] Mostafavi S. Ray D. Warde-Farley D. Grouios C. Morris Q. (2008). GeneMANIA: a real-time multiple association network integration algorithm for predicting gene function. Genome Biol. 9(Suppl. 1), S4 10.1186/gb-2008-9-s1-s4 18613948 PMC2447538

[B11] Peer D. Hacohen N. (2011). Principles and strategies for developing network models in cancer. Cell 144, 864–873 10.1016/j.cell.2011.03.001 21414479 PMC3082135

[B12] Warde-Farley D. Donaldson S. L. Comes O. Zuberi K. Badrawi R. Chao P. (2010). The GeneMANIA prediction server: biological network integration for gene prioritization and predicting gene function. Nucleic Acids Res. 38(Suppl. 2), W214–W220 10.1093/nar/gkq537 20576703 PMC2896186

[B13] Wu X. Huang H. Wei T. Pandey R. Reinhard C. Li S. D. (2012). Network expansion and pathway enrichment analysis towards biologically significant findings from microarrays. J. Integr. Bioinform. 9, 213 10.2390/biecoll-jib-2012-213 23079560

