# Supplementary material for: Ensemble inference by integrative cancer networks
Source: Front Genet. 2014 Mar 31;5:59. doi: 10.3389/fgene.2014.00059 (PMC3978335; doi:10.3389/fgene.2014.00059)
Supplement: Supplementary file 1 [file DataSheet1.ZIP › Data Sheet/Experiment.doc.docx]

Experimental Setting

Precisely, 2,5 uM 5-Aza-2’-deoxycytidine (5-Aza-dC), 300nM Trichostatin A (TSA) or both in combination were added to cell culture, and in the experiments where both drugs were used, the TSA was added to medium culture 12h after the 5-Aza-dC. The incubation period of cells culture was 24, 36, 48, 72 and 96 hours in the absence (control) or presence of drugs.

Microarray

cDNA microarray analysis was performed to provide expression measurements of 1920 genes of MDR-OS cells after the three different treatments. For each experimental point 10 ug of total RNA from a control and from the sample were labeled with Cy3 and Cy5 respectively, utilizing a 2-step aminoallyl labeling. Data analysis and statistics were carried out based on the ImaGene intensity values processed by the MAVI software (MWG Biotech AG), which solves saturation and calculates the normalization parameters. GeneSight 4.0 (BioDiscovery) was used to linearize the intensity values, calculate differential expression and perform gene-clustering analysis. Genes were selected as having at least 1.5-fold change in the log2 ratios of expression level and statistically significant (Wilcoxon test) at the 0.01 cutoff P value; Benjamini and Hochberg correction for multiple testing was applied. We used “R 3.0.1” (http://www.r-project.org/) for post-processing of the micro-array results. The figures were produced using igraph v. 0.6.6 (<http://cran.r-project.org/web/packages/igraph/index.html>).
