# Supplementary material for: Ensemble inference by integrative cancer networks
Source: Front Genet. 2014 Mar 31;5:59. doi: 10.3389/fgene.2014.00059 (PMC3978335; doi:10.3389/fgene.2014.00059)
Supplement: Supplementary file 1 [file DataSheet1.ZIP › Data Sheet/Supplementary Figure.docx]

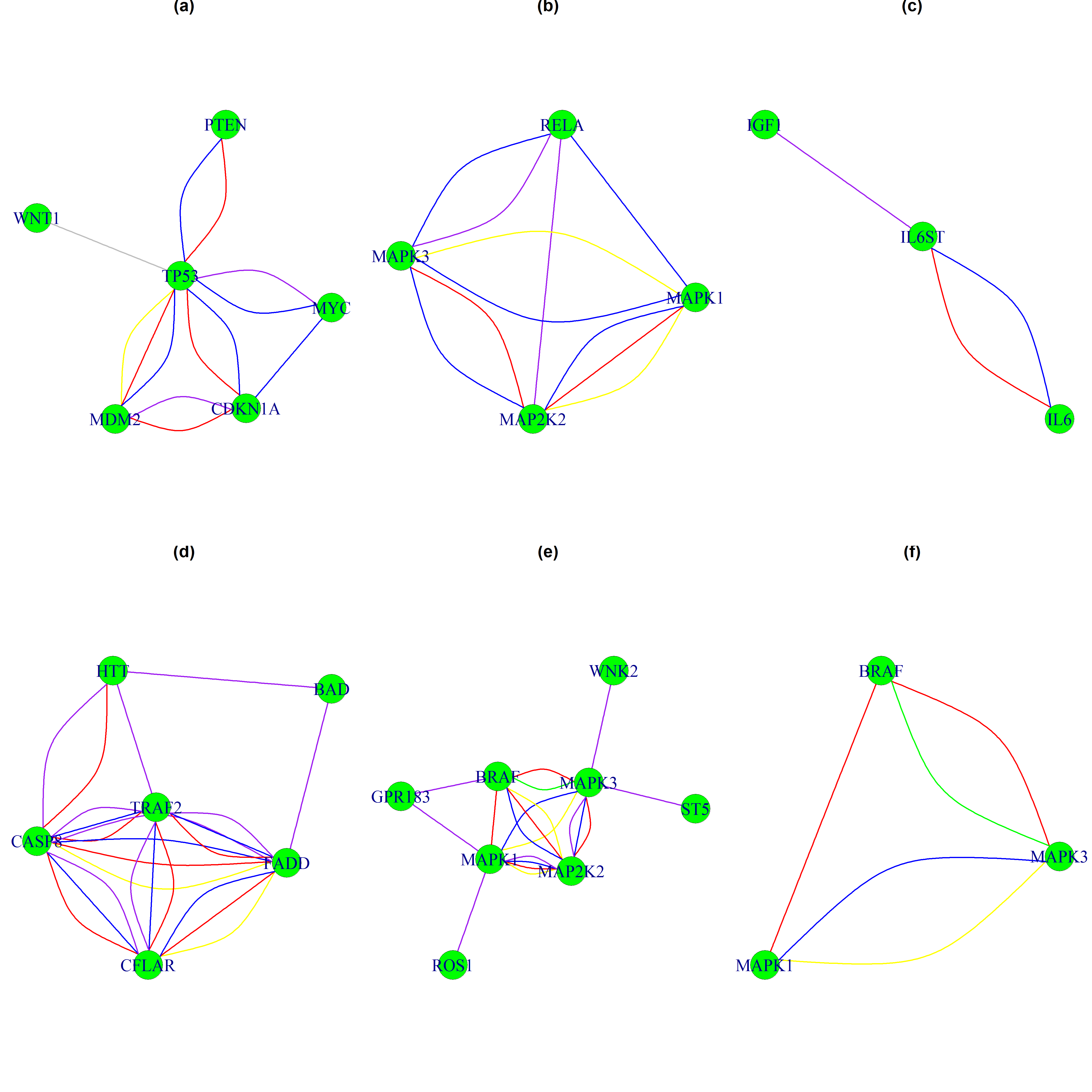


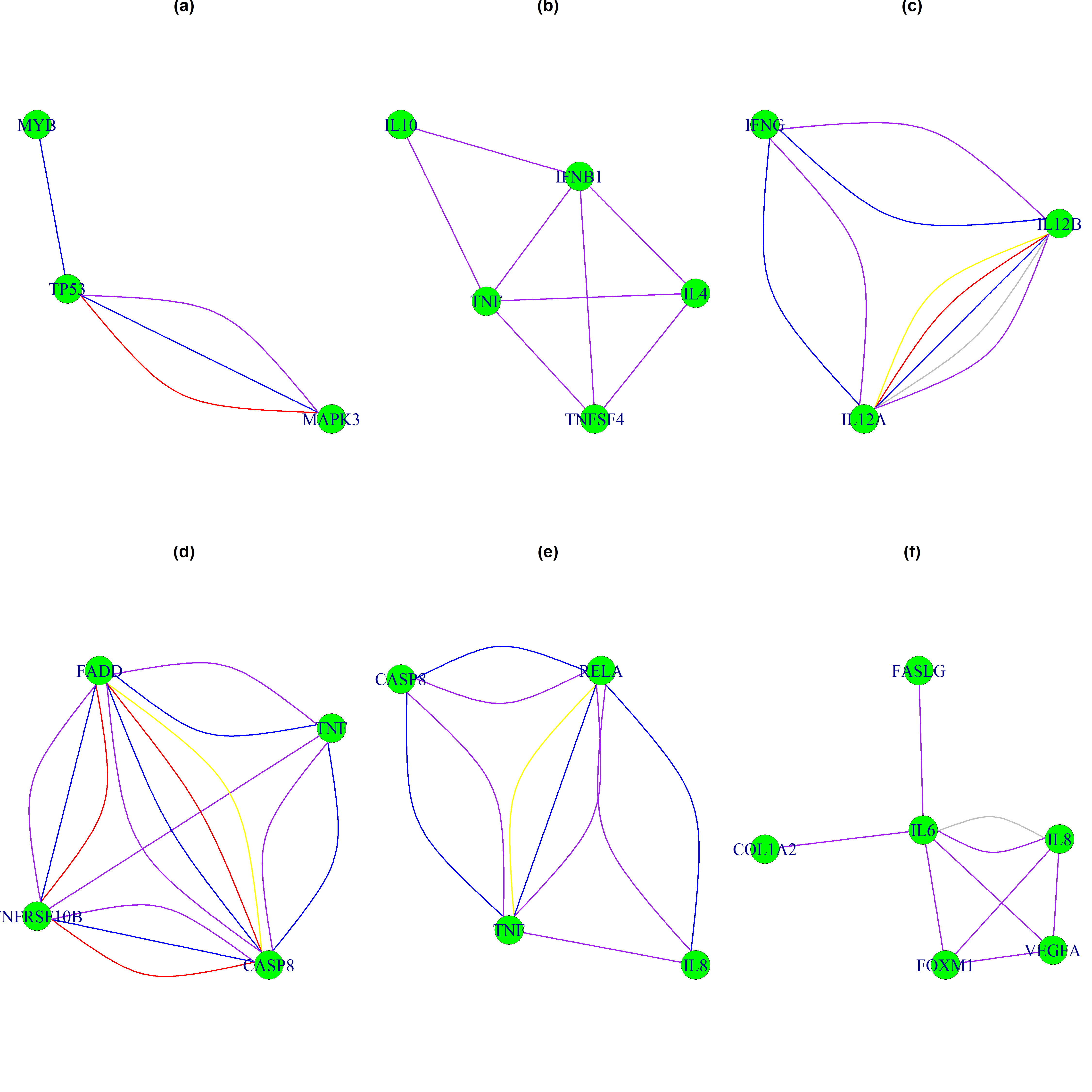


**Figure Legend.**

Examples of module markers involving multiple biological data types. Module markers are composed by gene markers enriched in one or more pathways. These genes are either DE genes or highly connected genes in an integrated network. The module markers reported form integrative sub-networks linked by interactions (red edges), pathways (blue edges), co-expression data (purple edges), genetic interactions (green edges), co-localization data (gray edges) and/or predicted interactions (yellow edges).

***Panel A (top)*** (a) Negative regulation of cell cycle process. (b) Toll-like receptor signaling pathway. (c) Positive regulation of osteoblast differentiation. (d) Extrinsic apoptotic signaling pathway. (e) ERK1 and ERK2 cascade. (f) Response to Epidermal Growth Factor stimulus.

The first three modules follow the DAC treatment, while the last three follow the TSA treatment.

***Panel B (bottom).*** (a) Positive regulation of histone modification: This is a marker mainly originated on pathway data. (b) Cytokine production involved in immune response: This is a marker completely originated on co-expression data. (c) Regulation of smooth muscle cell proliferation and regulation of cell killing: From co-expression and pathway data. One edge includes additional support. (d) Extrinsic apoptotic signaling pathway: Same as “c”. (e) Response to tumor necrosis factor: Same as “c” and “d”. (f) Blood vessel development: Originated in co-expression data. The first module corresponds to DAC treatment while the other five correspond to the co-treatment.
