# Supplementary figures and images for: Ensemble inference by integrative cancer networks
Source: Front Genet. 2014 Mar 31;5:59. doi: 10.3389/fgene.2014.00059 (PMC3978335; doi:10.3389/fgene.2014.00059)

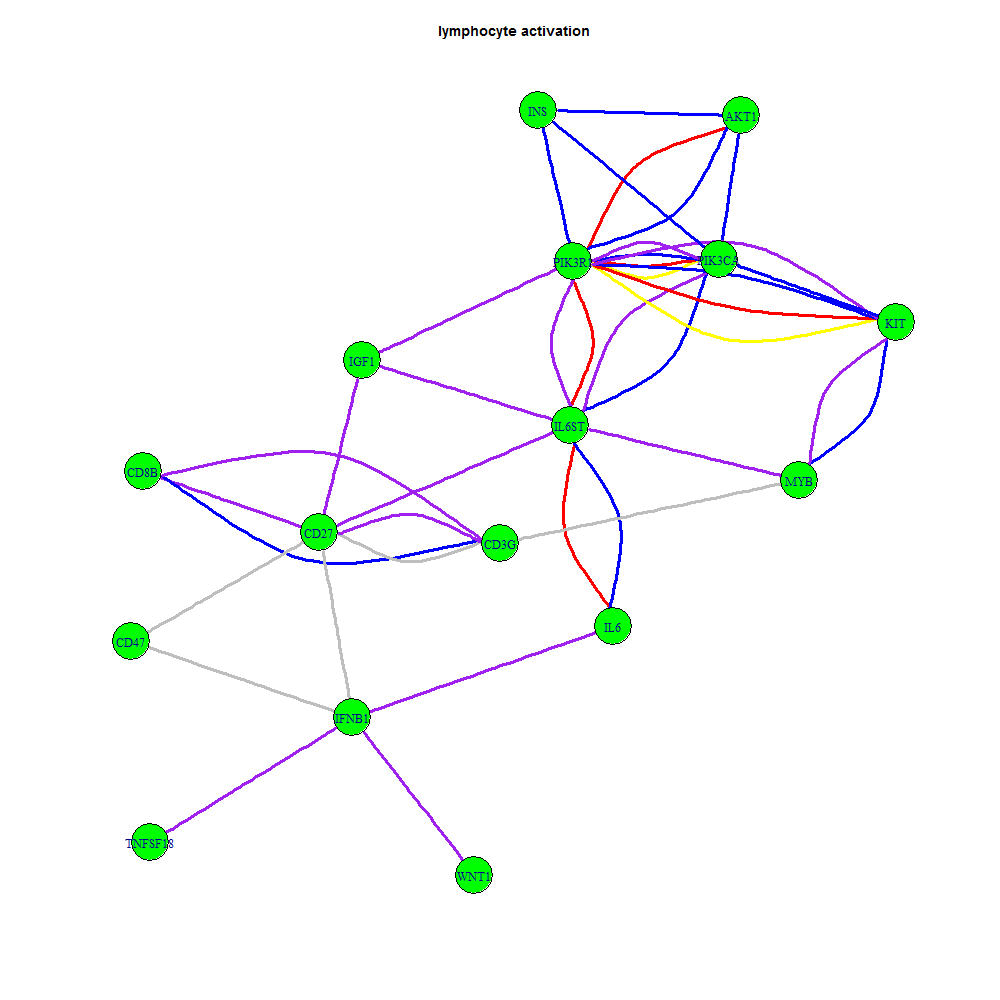

Supplement: Supplementary file 1 [file DataSheet1.ZIP › Data Sheet/module examples/module_DAC_11.png]

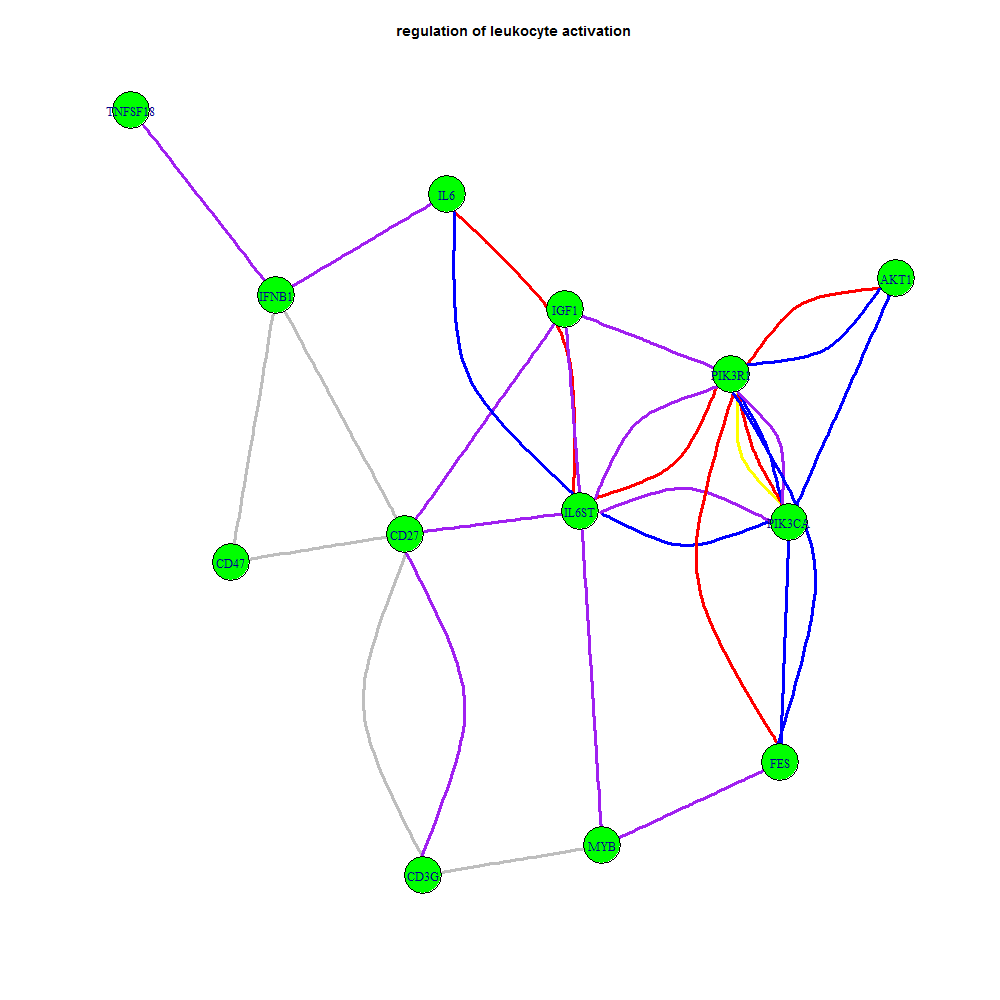

Supplement: Supplementary file 1 [file DataSheet1.ZIP › Data Sheet/module examples/module_DAC_17.png]

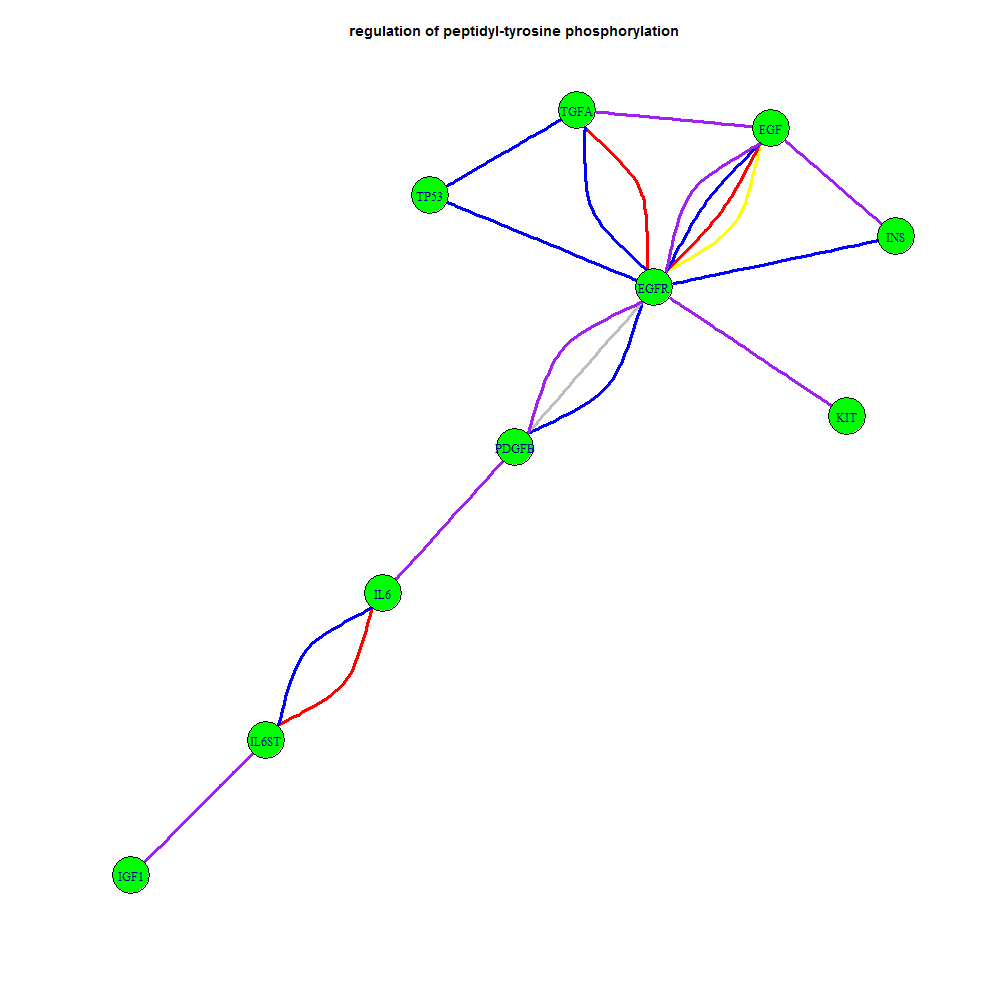

Supplement: Supplementary file 1 [file DataSheet1.ZIP › Data Sheet/module examples/module_DAC_28.png]

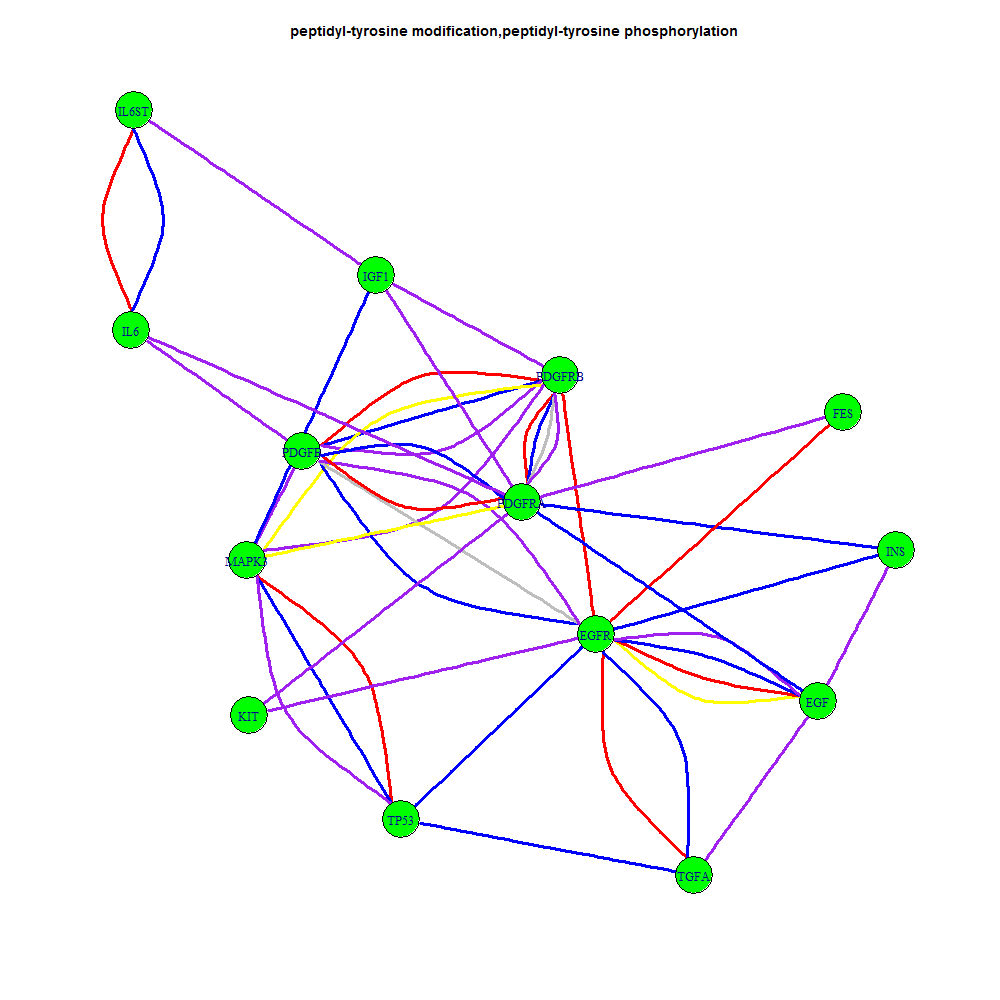

Supplement: Supplementary file 1 [file DataSheet1.ZIP › Data Sheet/module examples/module_DAC_4.png]

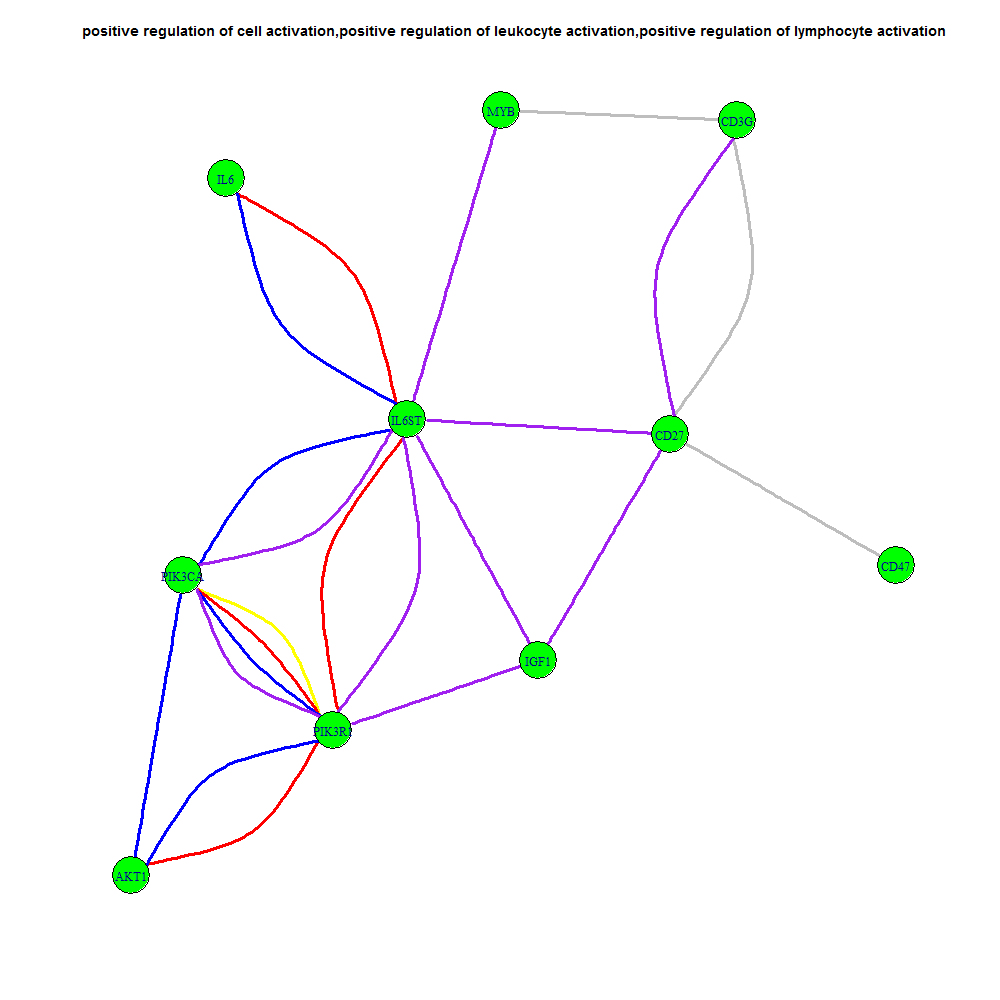

Supplement: Supplementary file 1 [file DataSheet1.ZIP › Data Sheet/module examples/module_DAC_45.png]

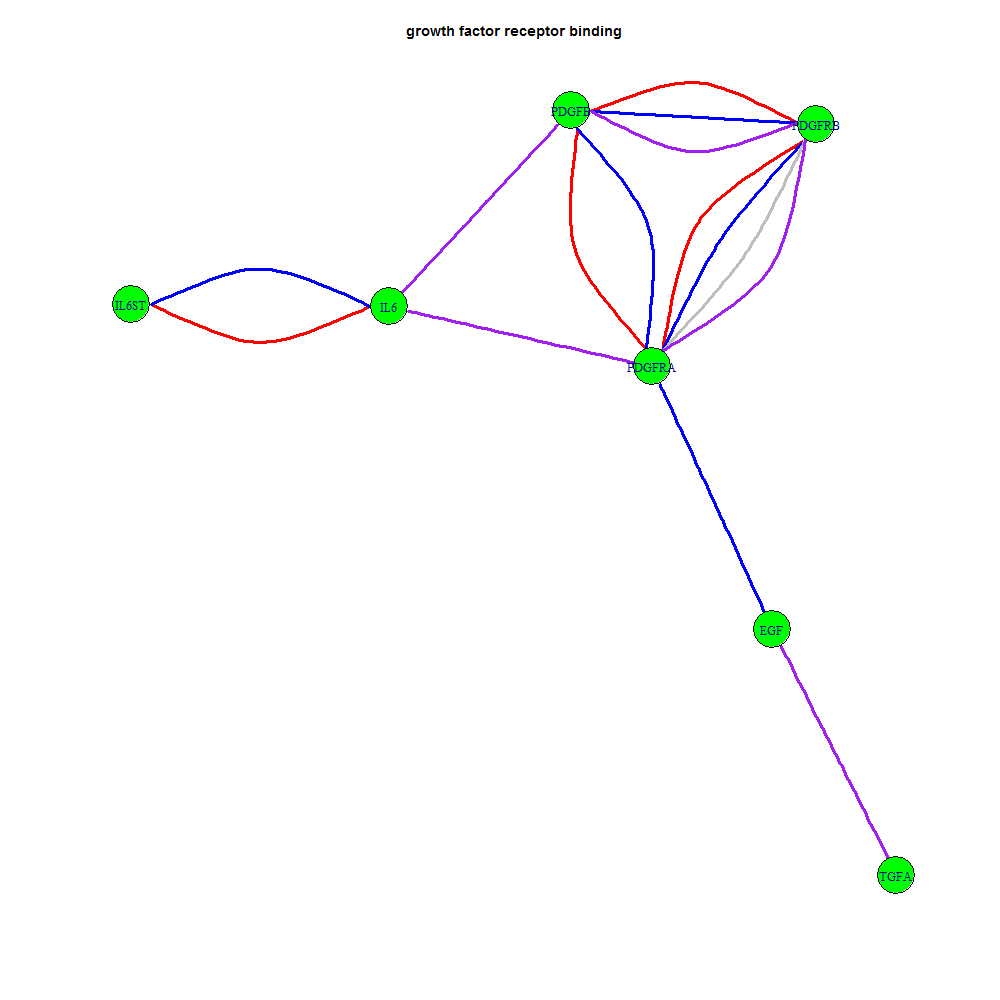

Supplement: Supplementary file 1 [file DataSheet1.ZIP › Data Sheet/module examples/module_DAC_52.png]

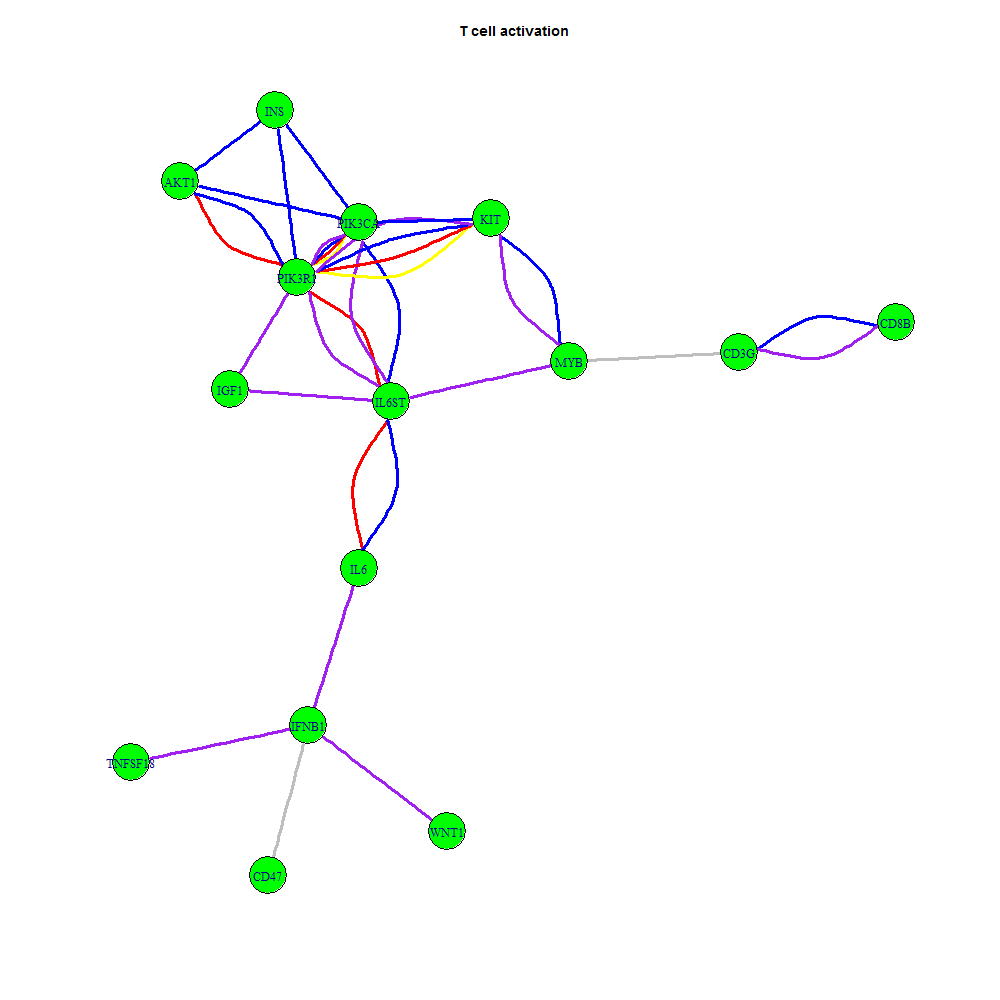

Supplement: Supplementary file 1 [file DataSheet1.ZIP › Data Sheet/module examples/module_DAC_7.png]

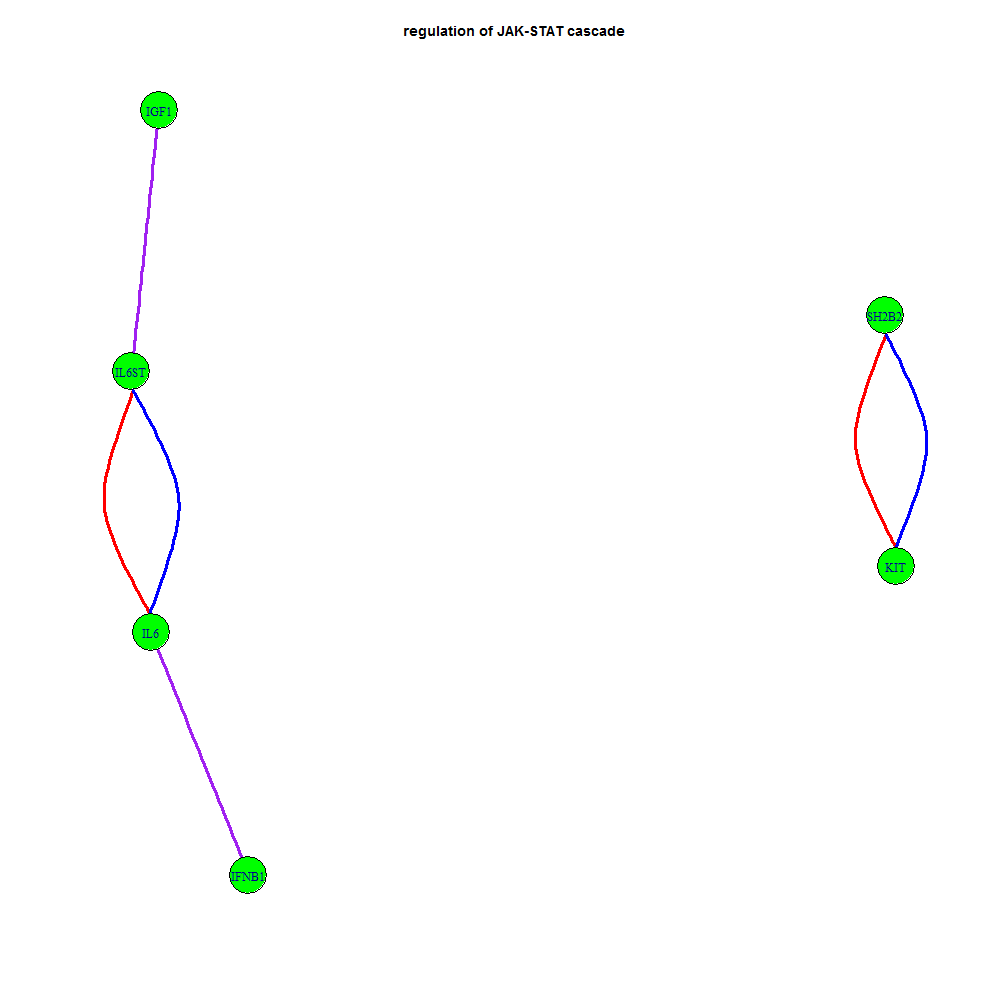

Supplement: Supplementary file 1 [file DataSheet1.ZIP › Data Sheet/module examples/module_DAC_73.png]

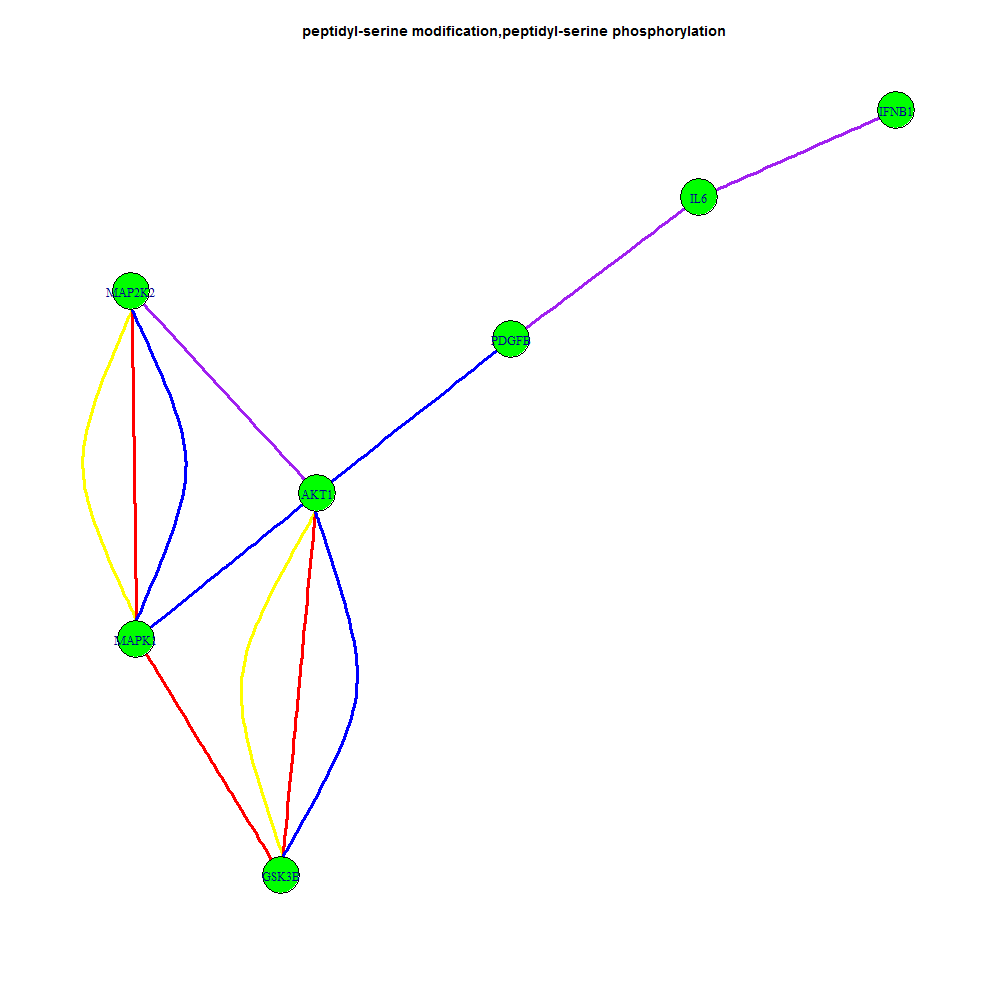

Supplement: Supplementary file 1 [file DataSheet1.ZIP › Data Sheet/module examples/module_DAC_81.png]

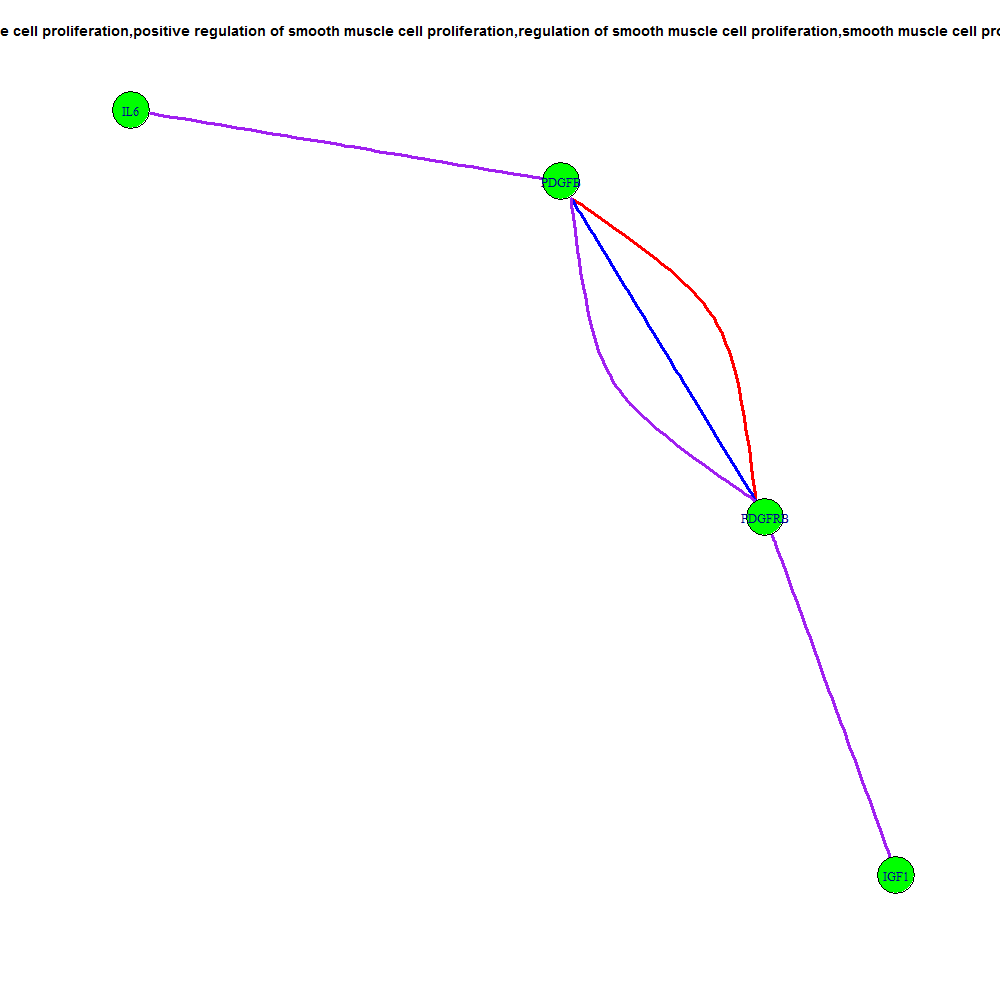

Supplement: Supplementary file 1 [file DataSheet1.ZIP › Data Sheet/module examples/module_DAC_83.png]

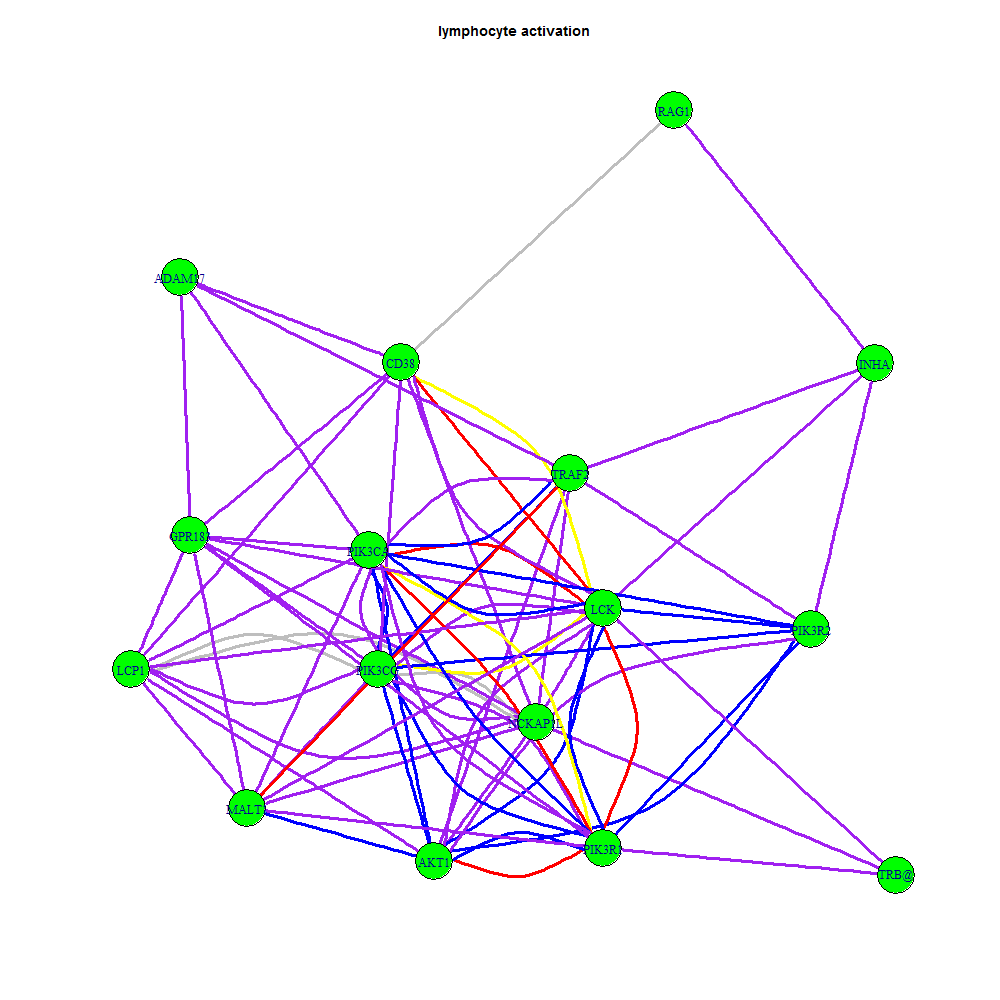

Supplement: Supplementary file 1 [file DataSheet1.ZIP › Data Sheet/module examples/module_TSA_12.png]

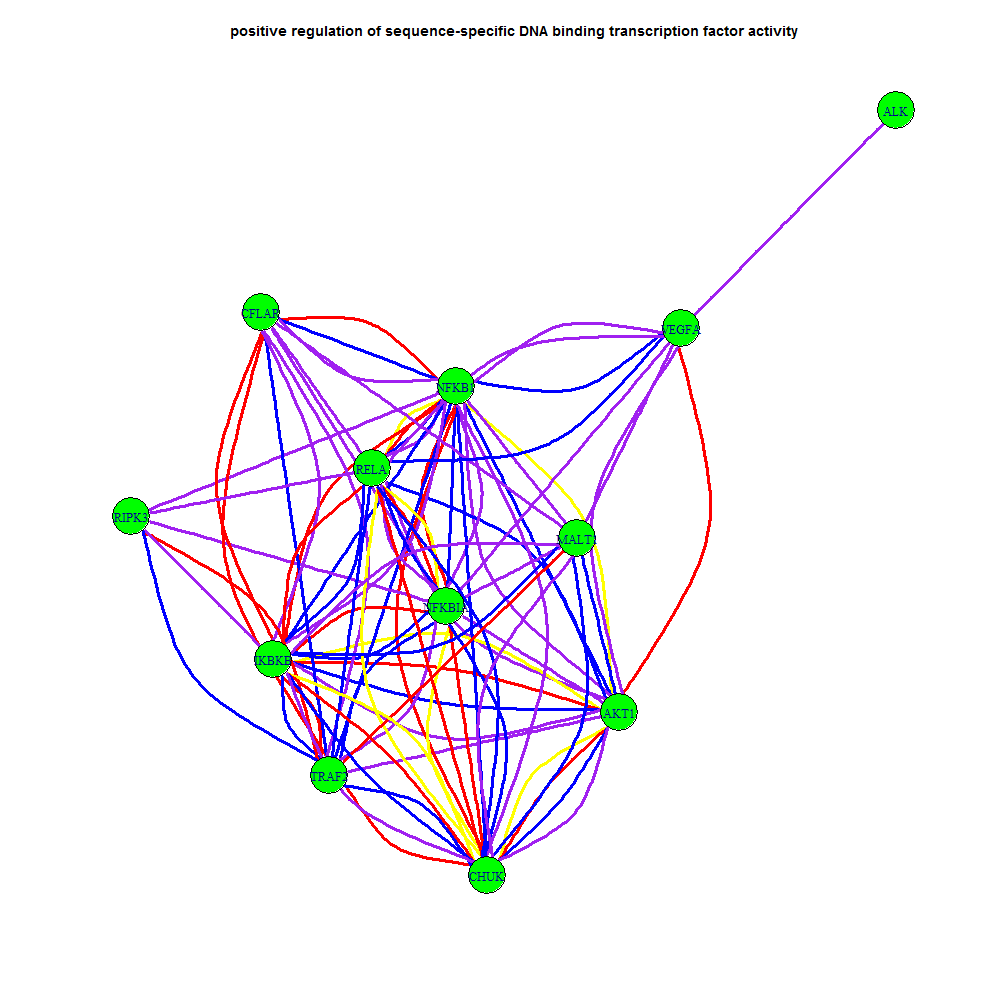

Supplement: Supplementary file 1 [file DataSheet1.ZIP › Data Sheet/module examples/module_TSA_17.png]

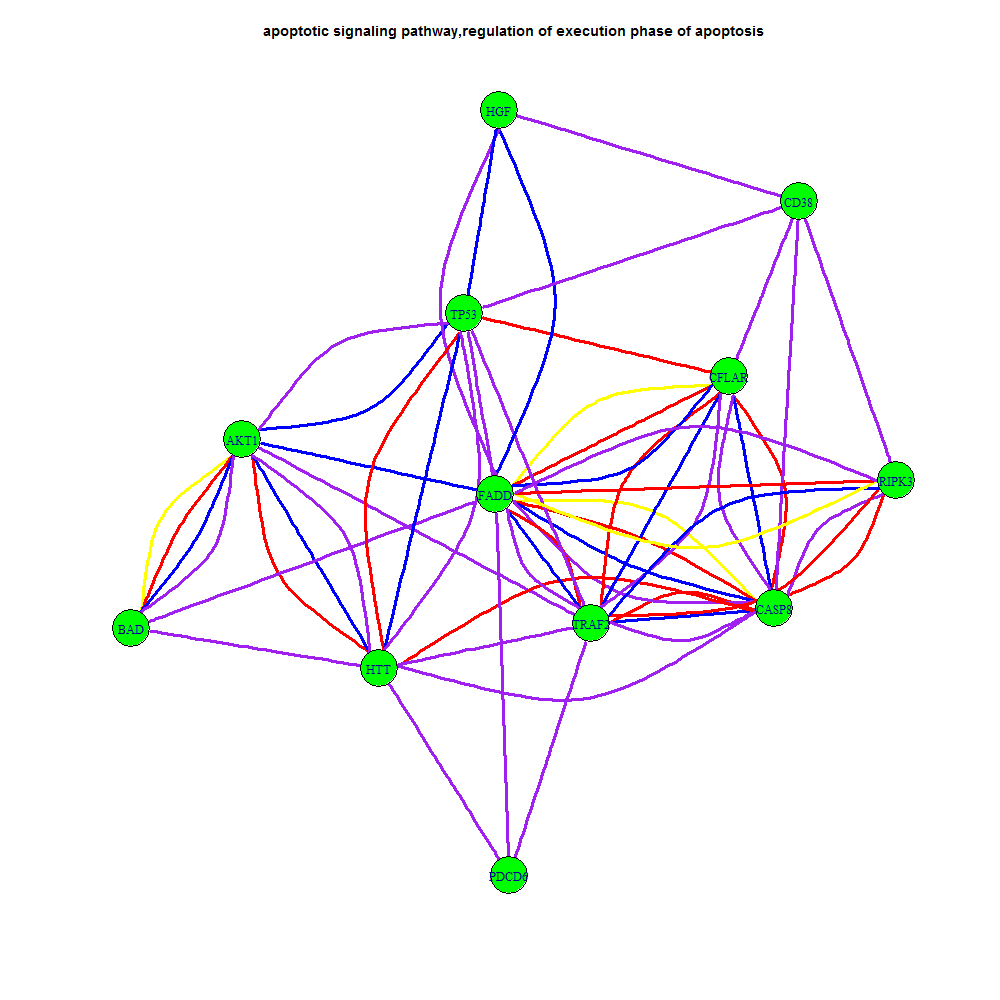

Supplement: Supplementary file 1 [file DataSheet1.ZIP › Data Sheet/module examples/module_TSA_21.png]

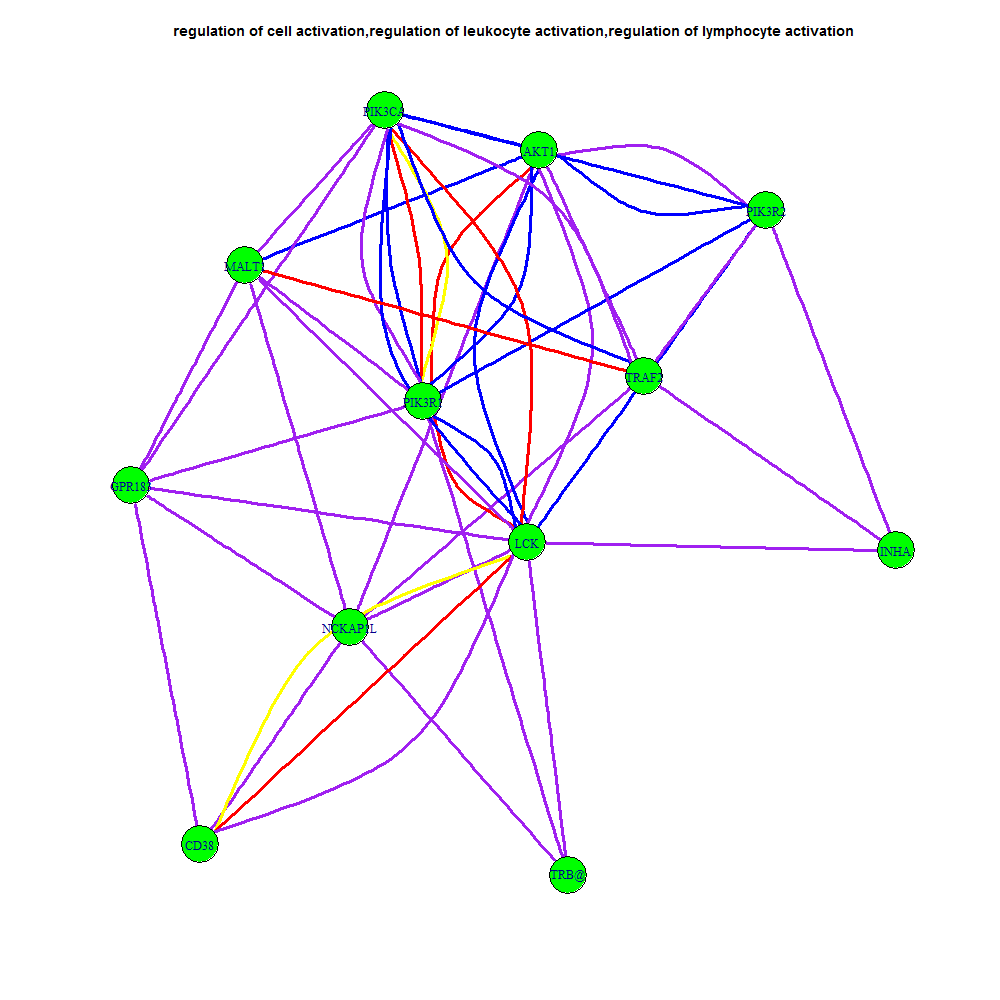

Supplement: Supplementary file 1 [file DataSheet1.ZIP › Data Sheet/module examples/module_TSA_28.png]

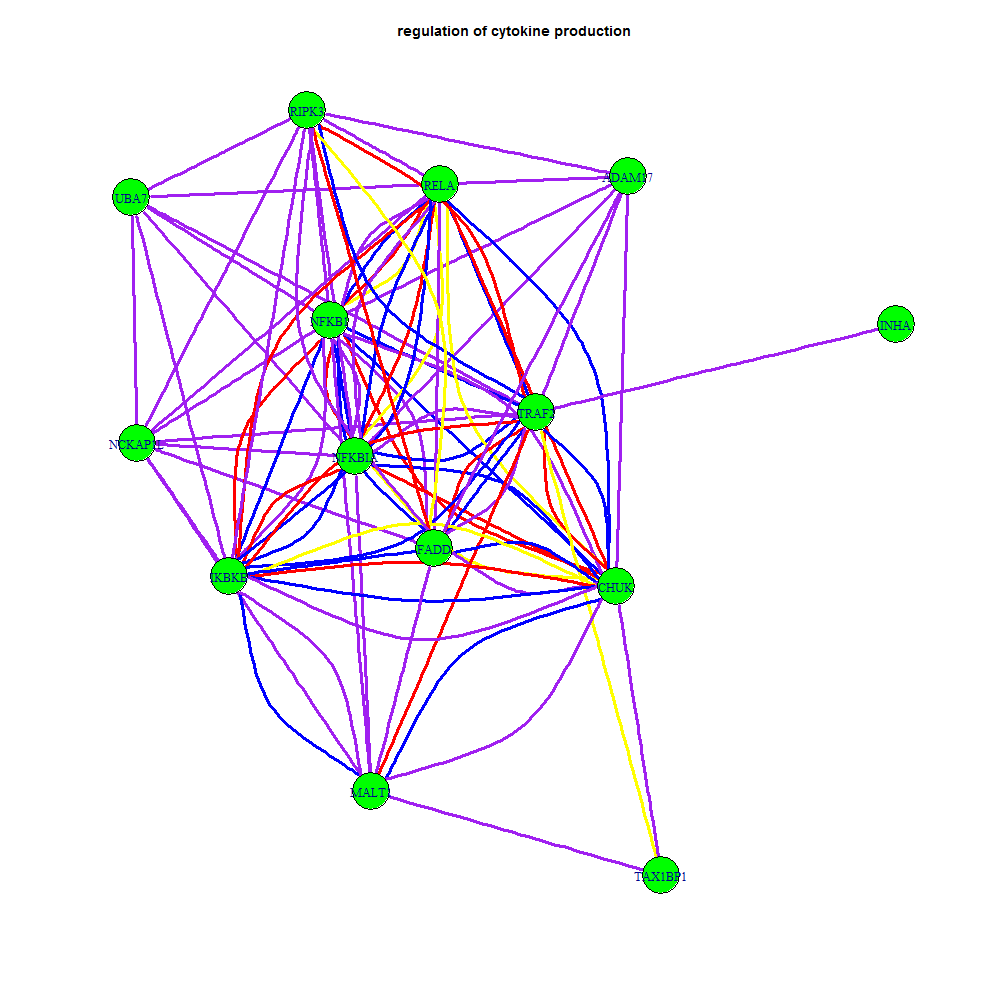

Supplement: Supplementary file 1 [file DataSheet1.ZIP › Data Sheet/module examples/module_TSA_29.png]

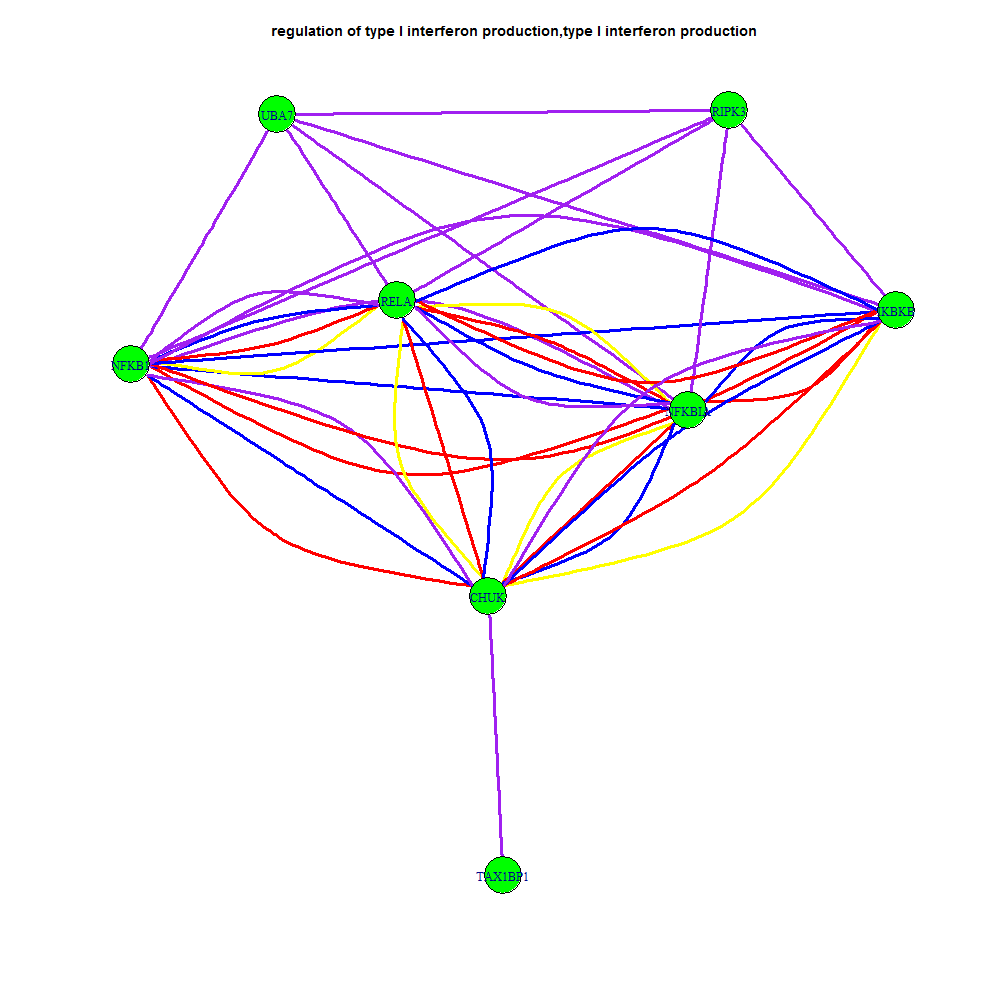

Supplement: Supplementary file 1 [file DataSheet1.ZIP › Data Sheet/module examples/module_TSA_32.png]

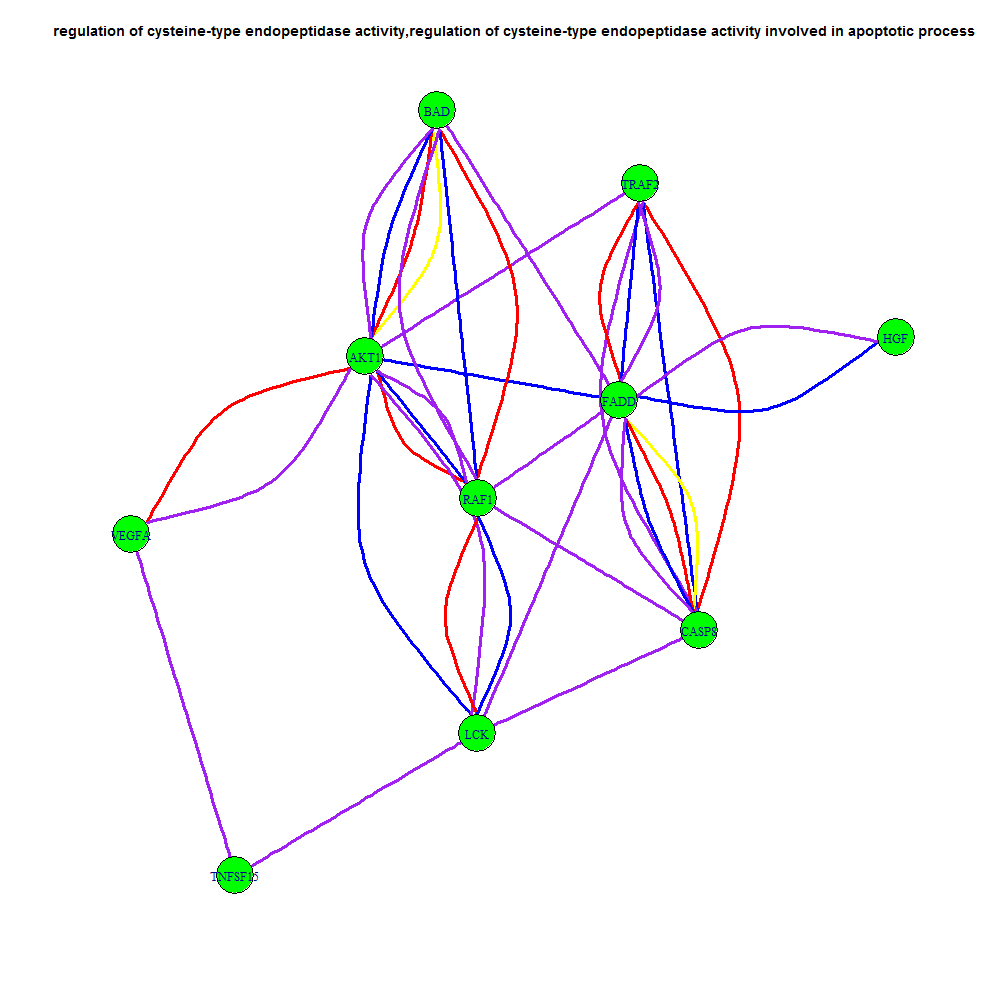

Supplement: Supplementary file 1 [file DataSheet1.ZIP › Data Sheet/module examples/module_TSA_33.png]

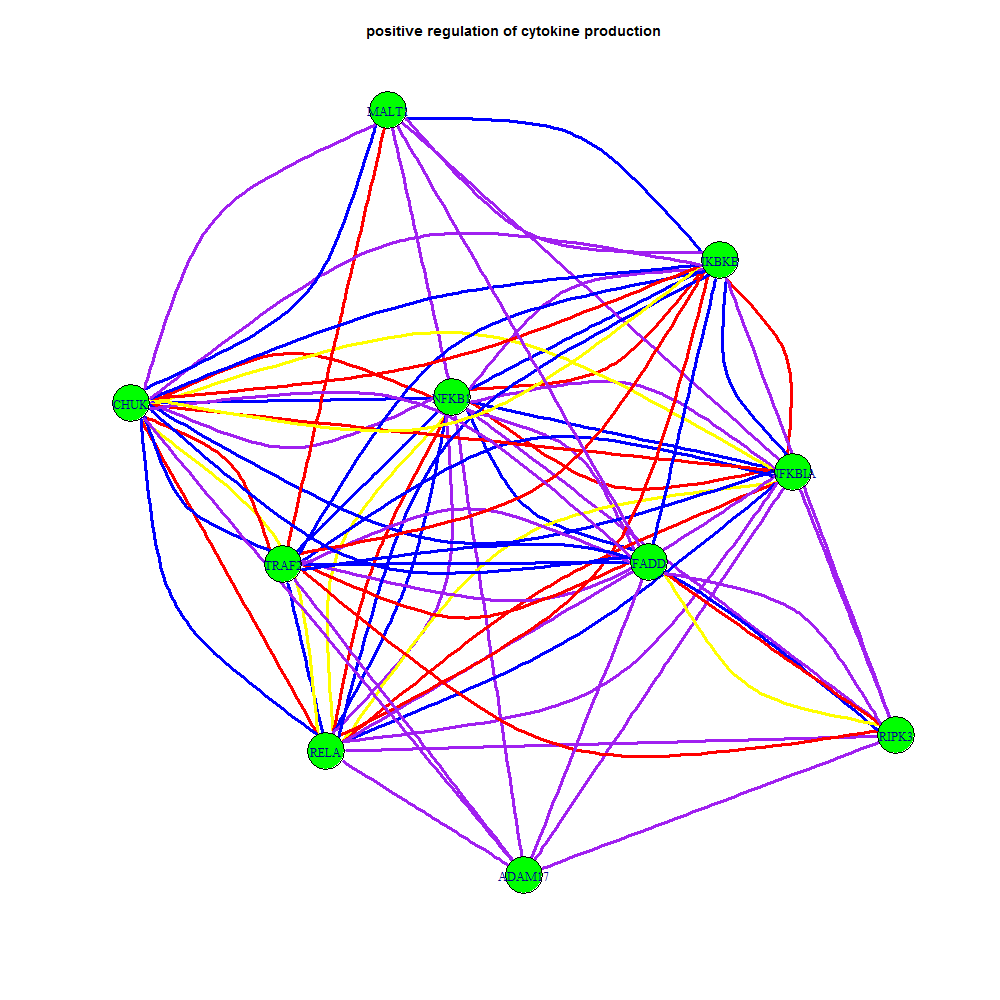

Supplement: Supplementary file 1 [file DataSheet1.ZIP › Data Sheet/module examples/module_TSA_34.png]

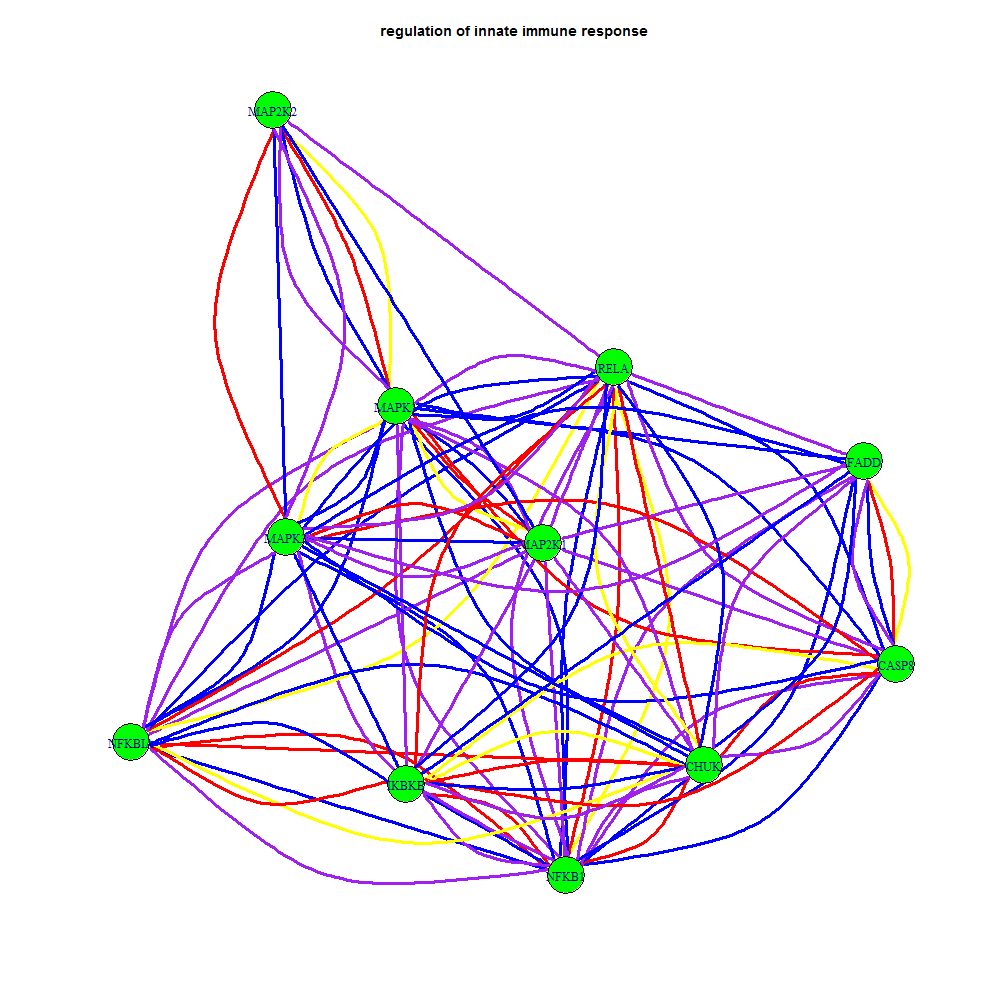

Supplement: Supplementary file 1 [file DataSheet1.ZIP › Data Sheet/module examples/module_TSA_36.png]
