# Supplementary material for: Ensemble inference by integrative cancer networks
Source: Front Genet. 2014 Mar 31;5:59. doi: 10.3389/fgene.2014.00059 (PMC3978335; doi:10.3389/fgene.2014.00059)
Supplement: Supplementary file 1 [file DataSheet1.ZIP › Data Sheet/Supplementary Table.doc]

**Supplementary Table – Part A.**

Module markers computed after DAC treatment, and found in co-treatment DAC-TSA.

| **Module Marker ID** | **# Pathways enriched in Module** | **Best Pathway** | **FDR –Best pathway** | **# Genes in Module Marker** | **# Genes in Pathways** | **Genes** |
| --- | --- | --- | --- | --- | --- | --- |
| 4 | 2 | peptidyl-tyrosine modification | 2.11e-08 | 14 | 156 | EGF, EGFR, FES, IGF1, IL6, IL6ST, INS, KIT, MAPK3, PDGFB, PDGFRA, PDGFRB, TGFA, TP53 |
| 7 | 1 | T cell activation | 9.97e-08 | 15 | 217 | AKT1, CD3G, CD47, CD8B, IFNB1, IGF1, IL6, IL6ST, INS, KIT, MYB, PIK3CA, PIK3R1, TNFSF18, WNT1 |
| 8 | 1 | regulation of cell activation | 3.48e-07 | 15 | 240 | AKT1, CD27, CD3G, CD47, FES, IFNB1, IGF1, IL6, IL6ST, MYB, PDGFB, PDGFRA, PIK3CA, PIK3R1, TNFSF18 |
| 11 | 1 | lymphocyte activation | 5.66e-07 | 16 | 294 | AKT1, CD27, CD3G, CD47, CD8B, IFNB1, IGF1, IL6, IL6ST, INS, KIT, MYB, PIK3CA, PIK3R1, TNFSF18, WNT1 |
| 13 | 1 | JAK-STAT cascade | 1.04e-06 | 10 | 89 | EGF, IGF1, IL6, IL6ST, KIT, MAPK1, MAPK3, SH2B2, STAT5A, STAT5B |
| 17 | 1 | regulation of leukocyte activation | 7.18e-06 | 13 | 221 | AKT1, CD27, CD3G, CD47, FES, IFNB1, IGF1, IL6, IL6ST, MYB, PIK3CA, PIK3R1, TNFSF18 |
| 24 | 1 | regulation of lymphocyte activation | 1.36e-05 | 12 | 198 | AKT1, CD27, CD3G, CD47, IFNB1, IGF1, IL6, IL6ST, MYB, PIK3CA, PIK3R1, TNFSF18 |
| 27 | 1 | regulation of T cell activation | 1.45e-05 | 11 | 162 | AKT1, CD3G, CD47, IFNB1, IGF1, IL6, IL6ST, MYB, PIK3CA, PIK3R1, TNFSF18 |
| 28 | 1 | regulation of peptidyl-tyrosine phosphorylation | 1.45e-05 | 10 | 125 | EGF, EGFR, IGF1, IL6, IL6ST, INS, KIT, PDGFB, TGFA, TP53 |
| 33 | 1 | positive regulation of peptidyl-tyrosine phosphorylation | 1.61e-05 | 9 | 97 | EGF, IGF1, IL6, IL6ST, INS, KIT, PDGFB, TGFA, TP53 |
| 40 | 1 | protein localization to nucleus | 2.56e-05 | 11 | 177 | AKT1, CD27, DAG1, EGF, EGFR, GSK3B, IGF1, IL6, MDM2, OPRD1, TOPORS |
| 44 | 3 | nuclear import | 5.69e-05 | 10 | 153 | AKT1, CD27, DAG1, EGF, EGFR, GSK3B, HTATIP2, IGF1, IL6, OPRD1 |
| 45 | 3 | positive regulation of lymphocyte activation | 5.69e-05 | 10 | 153 | AKT1, CD27, CD3G, CD47, IGF1, IL6, IL6ST, MYB, PIK3CA, PIK3R1 |
| 51 | 1 | positive regulation of T cell activation | 1.26e-04 | 9 | 130 | AKT1, CD3G, CD47, IGF1, IL6, IL6ST, MYB, PIK3CA, PIK3R1 |
| 52 | 1 | growth factor receptor binding | 2.03e-04 | 7 | 70 | EGF, IL6, IL6ST, PDGFB, PDGFRA, PDGFRB, TGFA |
| 60 | 3 | protein import into nucleus | 3.63e-04 | 9 | 150 | AKT1, CD27, DAG1, EGF, EGFR, GSK3B, IGF1, IL6, OPRD1 |
| 70 | 1 | immune effector process | 8.60e-04 | 11 | 268 | BNIP3L, CD27, CD8B, FES, IFNB1, IL6, INS, KIT, MYB, RELA, TNFSF18 |
| 73 | 1 | regulation of JAK-STAT cascade | 9.39e-04 | 6 | 60 | IFNB1, IGF1, IL6, IL6ST, KIT, SH2B2 |
| 74 | 2 | apoptotic signaling pathway | 9.42e-04 | 9 | 173 | AKT1, CASP9, CDKN1A, IGF1, PDCD6, SFN, TOPORS, TP53, TP73 |
| 81 | 2 | peptidyl-serine phosphorylation | 1.33e-03 | 7  7 | 99  104 | AKT1, GSK3B, IFNB1, IL6, MAP2K2, MAPK1, PDGFB |
| 83 | 4 | regulation of smooth muscle cell proliferation | 1.15e-02 | 4 | 35 | IGF1, IL6, PDGFB, PDGFRB |
| 95 | 1 | positive regulation of sequence-specific DNA binding transcription factor activity | 2.69e-03 | 8 | 156 | AKT1, IL6, INS, KIT, PTEN, RELA, TNFSF18, WNT1 |
| 122 | 1 | cytokine receptor binding | 7.88e-03 | 7 | 139 | CXCL13, ENG, IFNB1, IL6, IL6ST, PIK3R1, TNFSF18 |
| 127 | 4 | regulation of cysteine-type endopeptidase activity involved in apoptotic process | 8.46e-03 | 7 | 142 | AKT1, CASP9, CD27, IFNB1, MYC, SFN, XDH |

**Supplementary Table – Part B.**

Module markers computed after TSA treatment, and found in co-treatment DAC-TSA.

| **Module Marker ID** | **# Pathways enriched in Module** | **Best Pathway** | **FDR –Best pathway** | **# Genes in Module Marker** | **# Genes in Pathways** | **Genes** |
| --- | --- | --- | --- | --- | --- | --- |
| 12 | 1 | lymphocyte activation | 7.58e-08 | 16 | 294 | ADAM17, AKT1, CD38, GPR183, INHA, LCK, LCP1, MALT1, NCKAP1L, PIK3CA, PIK3CG, PIK3R1, PIK3R2, RAG1, TRAF2, TCRB |
| 17 | 1 | positive regulation of sequence-specific DNA binding transcription factor activity | 3.03e-07 | 12 | 156 | AKT1, ALK, CFLAR, CHUK, IKBKB, MALT1, NFKB1, NFKBIA, RELA, RIPK3, TRAF2, VEGFA |
| 18 | 9 | TRIF-dependent toll-like receptor signaling pathway | 3.27e-07 | 9 | 67 | CHUK, IKBKB, MAP2K1, MAP2K2, MAPK1, MAPK3, NFKB1, NFKBIA, RELA |
| 21 | 2 | apoptotic signaling pathway | 7.05e-07 | 12 | 173 | AKT1, BAD, CASP8, CD38, CFLAR, FADD, HGF, HTT, PDCD6, RIPK3, TP53, TRAF2 |
| 22 | 1 | T cell activation | 7.98e-07 | 13 | 217 | ADAM17, AKT1, LCK, LCP1, MALT1, NCKAP1L, PIK3CA, PIK3CG, PIK3R1, PIK3R2, RAG1, TRAF2, TCRB |
| 27 | 3 | positive regulation of lymphocyte activation | 1.84e-06 | 11 | 153 | AKT1, CD38, GPR183, LCK, MALT1, NCKAP1L, PIK3CA, PIK3R1, PIK3R2, TRAF2, TCRB |
| 28 | 3 | regulation of lymphocyte activation | 2.36e-06 | 12 | 198 | AKT1, CD38, GPR183, INHA, LCK, MALT1, NCKAP1L, PIK3CA, PIK3R1, PIK3R2, TRAF2, TCRB |
| 29 | 1 | regulation of cytokine production | 2.76e-06 | 14 | 296 | ADAM17, CHUK, FADD, IKBKB, INHA, MALT1, NCKAP1L, NFKB1, NFKBIA, RELA, RIPK3, TAX1BP1, TRAF2, UBA7 |
| 30 | 5 | positive regulation of innate immune response | 2.02e-05 | 10 | 156 | CASP8, CHUK, IKBKB, MAP2K1, MAP2K2, MAPK1, MAPK3, NFKB1, NFKBIA, RELA |
| 32 | 2 | regulation of type I interferon production | 3.90e-06 | 8 | 67 | CHUK, IKBKB, NFKB1, NFKBIA, RELA, RIPK3, TAX1BP1, UBA7 |
| 33 | 2 | regulation of cysteine-type endopeptidase activity involved in apoptotic process | 8.80e-06 | 10 | 142 | AKT1, BAD, CASP8, FADD, HGF, LCK, RAF1, TNFSF15, TRAF2, VEGFA |
| 34 | 1 | positive regulation of cytokine production | 2.52e-05 | 10 | 160 | ADAM17, CHUK, FADD, IKBKB, MALT1, NFKB1, NFKBIA, RELA, RIPK3, TRAF2 |
| 36 | 1 | regulation of innate immune response | 3.28e-05 | 11 | 210 | CASP8, CHUK, FADD, IKBKB, MAP2K1, MAP2K2, MAPK1, MAPK3, NFKB1, NFKBIA, RELA |
| 37 | 2 | positive regulation of T cell activation | 4.58e-05 | 9 | 130 | AKT1, LCK, MALT1, NCKAP1L, PIK3CA, PIK3R1, PIK3R2, TRAF2, TCRB |
| 38 | 2 | I-kappaB kinase/NF-kappaB cascade | 8.92e-05 | 10 | 186 | BMP7, CASP10, CASP8, CFLAR, CHUK, FADD, IKBKB, MALT1, NFKBIA, RELA |
| 41 | 2 | regulation of endopeptidase activity | 5.18e-05 | 11 | 221 | AKT1, BAD, CASP8, FADD, HGF, LCK, RAF1, SPINK1, TNFSF15, TRAF2, VEGFA |
| 48 | 1 | positive regulation of I-kappaB kinase/NF-kappaB cascade | 4.08e-04 | 8 | 128 | CASP10, CASP8, CFLAR, CHUK, FADD, IKBKB, MALT1, RELA |
| 56 | 2 | peptidyl-tyrosine modification | 1.59e-03 | 8 | 156 | ADAM17, DYRK1A, EGFR, HGF, MAPK3, TGFA, TP53, VEGFA |
| 57 | 2 | cellular response to mechanical stimulus | 1.64e-03 | 5 | 43 | BAD, CASP8, FADD, MAPK3, NFKB1 |
| 62 | 6 | activation of cysteine-type endopeptidase activity involved in apoptotic process | 2.14e-03 | 6 | 78 | BAD, CASP8, FADD, LCK, TNFSF15, TRAF2 |
| 73 | 1 | B cell activation | 5.74e-03 | 6 | 95 | ADAM17, CD38, GPR183, INHA, NCKAP1L, RAG1 |
| 74 | 1 | leukocyte differentiation | 5.74e-03 | 8 | 192 | ADAM17, CASP8, GPR183, INHA, LCK, NCKAP1L, RAG1, VEGFA |
| 75 | 4 | nucleotide-binding oligomerization domain containing signaling pathway | 5.74e-03 | 4 | 29 | CASP8, CHUK, IKBKB, RELA |
| 80 | 2 | peptidyl-serine modification | 6.79e-03 | 6 | 99 | AKT1, BRAF, MAP2K2, MAPK1, RAF1, VEGFA |
| 82 | 2 | nucleocytoplasmic transport | 7.27e-03 | 9 | 260 | AGFG1, AKT1, BMP7, CBLB, EGFR, HTT, MALT1, NFKBIA, RAB23 |
| 84 | 1 | immune effector process | 8.77e-03 | 9 | 268 | ADAM17, FADD, GPR183, LCK, LCP1, MALT1, PIK3CG, RELA, TRAF2 |
